# Maternal and Child Sexual Abuse History: An Intergenerational Exploration of Children’s Adjustment and Maternal Trauma-Reflective Functioning

**DOI:** 10.3389/fpsyg.2019.01062

**Published:** 2019-05-14

**Authors:** Jessica L. Borelli, Chloe Cohen, Corey Pettit, Lina Normandin, Mary Target, Peter Fonagy, Karin Ensink

**Affiliations:** ^1^THRIVE Laboratory, Department of Psychological Science, University of California, Irvine, Irvine, CA, United States; ^2^Department of Psychology, Laval University, Quebec, QC, Canada; ^3^Division of Psychology and Language Sciences, University College London, London, United Kingdom

**Keywords:** childhood sexual abuse, parent-child relationship, internalizing symptoms, externalizing symptoms, reflective functioning

## Abstract

**Objective:** The aim of the current study was to investigate associations, unique and interactive, between mothers’ and children’s histories of childhood sexual abuse (CSA) and children’s psychiatric outcomes using an intergenerational perspective. Further, we were particularly interested in examining whether maternal reflective functioning about their own trauma (T-RF) was associated with a lower likelihood of children’s abuse exposure (among children of CSA-exposed mothers).

**Methods:** One hundred and eleven children (*M*_age_ = 9.53 years; 43 sexual abuse victims) and their mothers (*M*_age_ = 37.99; 63 sexual abuse victims) participated in this study. Mothers completed the Parent Development Interview (PDI), which yielded assessments of RF regarding their own experiences of abuse, and also reported on their children’s internalizing and externalizing symptoms.

**Results:** Children of CSA-exposed mothers were more likely to have experienced CSA. A key result was that among CSA-exposed mothers, higher maternal T-RF regarding their own abuse was associated with lower likelihood of child CSA-exposure. Mothers’ and children’s CSA histories predicted children’s internalizing and externalizing symptoms, such that CSA exposure for mother or child was associated with greater symptomatology in children.

**Conclusion:** The findings show that the presence of either maternal or child CSA is associated with more child psychological difficulties. Importantly in terms of identifying potential protective factors, maternal T-RF is associated with lower likelihood of CSA exposure in children of CSA-exposed mothers. We discuss these findings in the context of the need for treatments focusing on increasing T-RF in mothers and children in the context of abuse to facilitate adaptation and reduce the intergenerational risk.

## Introduction

Child sexual abuse (CSA) is a recognized and prevalent risk factor for psychopathology, affecting approximately 20% of girls and 8% of boys under the age of 18 (Pereda et al., 2009b), although rates vary across cultures and contexts (0–60% of girls and 0–50% of boys; Pereda et al., 2009a). CSA victims demonstrate a range of short- and long-term physical and mental health problems –60% of sexually abused children present with moderate to severe symptoms of psychopathology (Kendall-Tackett et al., 1993; Maniglio, 2009), over one third manifest clinically significant depressive symptoms (Mathews et al., 2013), and up to 47% develop externalizing problems (McCrae et al., 2006; Beaudoin et al., 2013; Mathews et al., 2013). Even as compared to other forms of abuse, the impacts of CSA may be especially noxious—CSA-exposed children report significantly higher psychological symptoms than children exposed to other forms of abuse (Lewis et al., 2016) and are also at greater risk for physical health problems (Irish et al., 2010), sexualized behavior, dissociation (Ensink et al., 2016b), and interpersonal relationship difficulties later in life (Maniglio, 2009; McCloskey, 2013) than non-abused children. With myriad adverse outcomes linked to CSA, it is crucial to consider potential factors relevant to the prevention of CSA in addition to treatment mechanisms that may buffer against these negative outcomes.

### CSA Across Generations

Childhood sexual abuse has impacts across familial generations in terms of the likelihood of sexual victimization (McCloskey, 2000). There is converging evidence of intergenerational patterns of risk and mothers who are CSA survivors are up to 3.6 times as likely to have children who are also exposed to sexual violence compared to mothers who were not abused (Oates et al., 1998; McCloskey, 2000; Testa et al., 2011; Wearick-Silva et al., 2014; Testoni et al., 2018). Indeed, links in CSA risk spanning three generations have even been documented (Leifer et al., 2004; McCloskey, 2013).

These studies do not implicate mothers as responsible for their children’s sexual victimization; rather, child outcomes are typically interpreted as the result of harmful environments and cascading risk factors associated with sexual abuse (e.g., early pregnancy, poverty, depression, substance use, posttraumatic stress symptoms) that could negatively impact parenting resources/abilities as well as increase the child’s risk for victimization (Serbin and Karp, 2004; Noll et al., 2009). Indeed, CSA-exposed mothers report significantly greater parenting stress (Cross, 2001; Macias, 2005), demonstrate less warmth toward their children, and show less overall skill in parenting than mothers without a history of CSA (Cohen, 1995; Barrett, 2009; Cross et al., 2016). In addition, CSA-exposed mothers are more likely to demonstrate role reversal, or to depend on children to fulfill their needs, and are more self-focused in parenting than non-CSA mothers, which may lead to more protective and parent-focused behaviors in their children (Burkett, 1991). However, CSA-exposure does not inevitably or always have negative implications for parenting. One study failed to find an association between CSA–exposure and parenting problems – working with a low risk community sample, Ensink and colleagues did not find evidence for an association between CSA-exposure and insensitive parenting (Ensink et al., 2017b) or disorganized infant attachment (Ensink et al., 2016c). However, CSA-exposed mothers report significantly more identity diffusion and primitive defenses, indicative of underlying risk mechanisms that may or may not manifest depending in their interaction with other risk factors.

Despite the documented associations between CSA exposure in childhood and difficulties parenting, widespread awareness of the risk associated with parents’ CSA exposure is not acknowledged (Testoni et al., 2018), with few programs existing to support this population of parents.

### Associations With Psychopathology

As described above, ample research demonstrates that CSA exposure is associated with risk for psychopathology well into adulthood (e.g., Mathews et al., 2013). In addition to this direct link between CSA and psychopathology, there are also reasons to suspect that CSA exposure among mothers may be associated with psychopathology risk in their children. We base this supposition on research documenting links between CSA exposure and parents’ behavior, emotion, and cognition (e.g., Cross et al., 2016). Further, although not specific to CSA exposure, psychopathology rates are greater among children of parents exposed to a diverse array of traumatic experiences (Dekel and Goldblatt, 2008; Yehuda and Bierer, 2009; see Bowers and Yehuda, 2016, for a review). Taken together, these findings indicate that trauma in general, and CSA exposure in particular, may confer risk for disruptions in caregiving behavior, which in turn could confer risk for maladjustment in children.

Still, parental behavior as well as parental mentalizing may *also* serve a protective role in children’s development, reducing the likelihood of children being exposed to CSA and promoting adjustment in the face of children’s CSA exposure. These precise links have not been tested before in the literature, but we draw upon findings from related literatures in suggesting parental characteristics could be protective factors in the case of CSA specifically. In terms of the argument that parenting factors could reduce the likelihood of children’s abuse exposure among CSA-exposed parents, one study found that high levels of maternal warmth were associated with lower levels of children’s CSA exposure among women who themselves had been maltreated as children (Jaffee et al., 2013), a pattern echoed in the findings of a recent meta-analysis (Schofield et al., 2013).

### From Reflective Functioning to Trauma-Reflective Functioning

For decades, attachment researchers focused on the role of sensitive parental behavior in predicting children’s attachment security and emotional well-being, with relatively less attention paid to understanding the psychological capacities that underlie sensitivity. Our understanding of the psychological origins of parental sensitivity was advanced considerably by Fonagy and colleagues’ theorizing (Fonagy and Target, 1996; Fonagy et al., 2002), which posited that mentalization—the capacity to place oneself in the mind of others, imagining the intentions and reasons underlying others’ and being aware of one’s own reactions and their impact on others—may facilitate the enactment of sensitive responses to others’ emotional needs.

Since Fonagy’s initial theorizing (Fonagy et al., 1991), the field has witnessed a proliferation of mentalization research, a construct now operationalized using diverse methods of assessment (e.g., parental reflective functioning, Slade et al., 2005; mind-mindedness, Meins et al., 2003; insight, Oppenheim and Koren-Karie, 2013, adult reflective functioning, Fonagy et al., 1991; child reflective functioning, Ensink et al., 2015; adolescent reflective functioning, Ha et al., 2013). Studies demonstrate positive associations between mentalization, attachment security, emotion regulation, and mental health, both within individuals (e.g., Borelli et al., 2018a; Duval et al., 2018) and across parent-child dyads (e.g., Fonagy et al., 1991; Borelli et al., 2016). In the context of parenting, mentalization is regulating in the context of high levels of physiological arousal (Borelli et al., 2017). In the current investigation, we build upon work suggesting that parental reflective functioning (RF) is an essential capacity for providing sensitive care with infants (e.g., Slade et al., 2005; Suchman et al., 2010).

In addition to promoting self regulation and responsiveness to the child’s psychological experience under routine parenting circumstances, RF is particularly important in the wake of traumatic experiences (Allen et al., 2008; Fonagy and Luyten, 2009; Ensink et al., 2016a). Scholars contend that the capacity to mentalize may be at the core of resilience processes following early adversity, (Fonagy et al., 2002; Fonagy et al., 2003), enabling people to make sense of their own and others’ emotions and thought processes following these experiences. In support of this theorizing we have previously found higher parental RF and child RF to be associated with better child psychological adaptation after CSA (Ensink et al., 2016a, 2016b). Furthermore, research suggests that the children of parents with insecure attachment histories and histories of neglect or abuse are less likely to have insecure attachment themselves when their parents exhibit higher RF (Fonagy et al., 1991) and higher parental RF (Katznelson, 2014). Further, infants of mothers exposed to trauma or neglect are more likely to have secure attachment when their mothers have higher RF.

In a further step toward understanding the role of mentalizing in recovery from trauma, researchers learned that mentalizing specific to trauma, so-called Trauma-RF, was frequently markedly lower than RF regarding self and others (Ensink et al., 2015). In addition, higher Trauma-RF in parents was associated with a lower risk of infant attachment disorganization (Berthelot et al., 2015). An important missing piece of the puzzle is whether higher Trauma-RF in parents regarding their own past traumatic experiences is associated with reduced risk of their children being victimized. Trauma-RF may help parents with histories of trauma effectively use their cognitive and emotional resources to identify risk, as well as to regulate fear and vulnerability in the service of protecting children (Ensink et al., 2015). Conversely, when parents have low levels of Trauma-RF, this could result in dangerous blind spots for them (Fonagy, 1993). In this vein, there is preliminary evidence that when mothers are able to consider the effects of their own childhood victimization, measured through a lack of dissociation in recounting their trauma, their children’s risk for child abuse is reduced (Egeland and Susman-Stillman, 1996). Mentalizing and Trauma-RF are considered broader constructs than dissociation, with both having implications for recovery from trauma (see Ensink et al., 2015, 2017a; for a discussion). Thus, understanding the links between parents’ Trauma-RF in potentially attenuating the likelihood of abuse among children is important in enhancing our understanding of potential ways to interrupt the cycle of abuse, an important public health aim (Testoni et al., 2018). In the current study, we build upon this prior work in testing whether among CSA-exposed mothers, higher Trauma-RF regarding their own CSA is associated with lower odds of children having experienced CSA.

### Current Investigation

The aims of the current study are to explore the associations between mothers’ sexual abuse histories, children’s sexual abuse histories, mothers’ RF, and children’s clinical symptoms. We test a series of hypotheses regarding the interrelations of these constructs. First, based on demonstrated links showing intergenerational risk in CSA exposure (McCloskey, 2000), we hypothesize that children whose mothers have experienced CSA will be more likely to have CSA histories themselves (Hypothesis 1), a replication of prior findings. Next, focusing exclusively on CSA-exposed mothers, we predict that higher maternal Trauma-RF regarding mothers’ own CSA will be associated with a lower likelihood of children’s direct CSA exposure (Hypothesis 2). Here we note that although our theoretical model holds that maternal Trauma-RF may protect against children’s exposure to CSA, the cross-sectional nature of our study design does not enable us to conclude whether maternal Trauma-RF regarding their own CSA-exposure is causally related to children’s CSA-exposure or whether it is even temporally antecedent.

Then we explore the associations between CSA exposure, both direct (children’s) and vicarious (mothers’), and children’s internalizing and externalizing symptoms. Here we predict that when mothers or children have histories of CSA, children’s internalizing and externalizing symptoms will be higher than when neither party has experienced abuse (Hypothesis 3); in other words, Hypothesis 3 holds that direct or vicarious exposure to CSA will be associated with higher clinical symptoms in children.

## Materials and Methods

Sexually abused children and their mothers were referred to a university clinic in southern Quebec, Canada by doctors, social services, or mental health practitioners at community health services and hospitals in the city and surrounding regions. The community comparison group was recruited through advertisements at Community Health Services and schools through pamphlets soliciting participation in a study on the impact of CSA as part of a comparison group. The comparison group was selected to broadly match the sociodemographic, age (within 6 months), and gender characteristics of the abused group.

Of the total sample (*N* = 111), 43 children and 63 mothers had CSA exposure. The demographic features of the resulting sample are described in Table 1. More than half (61.3%) of the children were female and the average age of the participants was 9.53 years (*SD* = 1.45 months; age range = 7–12 years). Mothers in this sample were on average 37.99 years (*SD* = 6.20). Reflecting the demographics of the region, most participants were Caucasian (98%). Assessments took place at a university-affiliated child and adolescent consultation service. Parents received a modest stipend to cover transport costs and children were invited to choose a small toy as compensation for their participation.

**TABLE 1:** Sample characteristics as a function of abuse history.

	C No CSA		C CSA		M No CSA		M CSA		Entire Sample	
	*M/%*	*SD*	*M/%*	*SD*	*M/%*	*SD*	*M/%*	*SD*	*M/%*	*SD*
**Demographic**										
Child age in months	114.35	16.02	114.45	19.6	115	16.52	113.92	18.13	114.39	17.37
% > $25,000 annual income	31%		44%		23%		46%		36%	
% male children	41.20%		35%		40%		38%		39%	
% Caucasian children	91.20%		93%		90%		94%		93%	
**Clinical Symptoms**										
CBCL Internalizing	56.42	9.86	64.9	8.93	54.63	10.16	63.92	8.5	59.75	10.34
CBCL Externalizing	53.46	11.42	63.79	11.79	52.23	11.17	61.81	12.08	57.51	12.57

*C, child; M, mother.*

### Measures

#### Children’s Abuse History

Information regarding CSA was based on medical and social work reports and information from police inquiries, including statements of admission by the abuser. This is the standard legal protocol followed in Canada for the assessment of CSA. Thus, all children had legally documented cases of CSA and were referred to participate in the study because of having had this experience. To facilitate the comparison of this sample of CSA-exposed children to those assessed in other investigations, we provide additional information regarding the nature of the CSA experienced by the children, noting that these variables were not employed in hypothesis testing. Of the group of children exposed to CSA, 46% experienced CSA on two or three occasions, 36% experienced CSA on four or more occasions and 18.2% experienced CSA on one occasion. Approximately half of the perpetrators were family members, including fathers (60%), siblings (22%), and step-parents (18%). The other half of the children were abused by people who weren’t part of their immediate families, including acquaintances (67%) or by a member of the extended family (33%). A small minority (5%) had experienced violent sexual abuse and (23%) had vaginal or anal penetration with a penis or object. Slightly more than half (54%) first experienced CSA before 4 years of age and slightly more than half of the children (55%) denounced the CSA.

In contrast, parents of comparison group children were recruited from the community. To ensure that these participants were free of CSA, clinical researchers interviewed the mothers about the child’s developmental history and traumatic life events to ensure comparison sample children did not have CSA histories. For the purposes of this study, we classified children into two groups: those who had experienced sexual abuse, and those who had not.

#### Mothers’ Abuse History

Information regarding mothers’ own histories of childhood sexual abuse was based on self-report data gathered at the same time as other demographic measures during the study visit. Mothers were asked whether or not they had experienced sexual abuse as a child, whether this abuse was perpetrated by a family member or a non-family member, and they were asked to briefly describe the severity, length, and frequency of the abuse. For the purposes of this study, we classified mothers into two groups based on those who had experienced sexual abuse and those who had not.

#### Children’s Psychopathology Symptoms

The Child Behavior Checklist (CBCL) is a 118-item questionnaire used to assess a broad range of internalizing and externalizing difficulties (Achenbach, 1991). In the present study, we used the parent report internalizing and externalizing scale for children aged 6–18 (Achenbach and Rescorla, 2001). Parents rate behavior on a three-point scale as 0 (absent), 1 (occurs sometimes), or 2 (occurs often) for items such as, “breaks rules at home, school, or elsewhere.” The CBCL demonstrated good psychometric properties (Achenbach and Rescorla, 2001). In the current sample, Cronbach’s alphas indicated satisfactory internal consistency (αs ranged between 0.87 and 0.92).

#### Maternal Reflective Functioning

Maternal RF was measured using the Parent Development Interview-Revised (PDI-R; Slade et al., 2004). The PDI-R is a 45-item semi-structured interview developed to assess parental mentalizing regarding themselves as parents (“What gives you the most joy as a parent?”), their child [“Could you describe (name of the child)?”], and the parent-child relationship [“Describe a time when you became really angry with (name of child)”; “What effect did this have on him/her?”]. Reliability estimates using the coding manual are good, with ICCs ranging from 0.78 to 0.95 (Slade et al., 2005). For the purposes of this investigation, the researchers added questions regarding the mother’s reflections on her own CSA history (when reported), which were coded using the same scale as all other questions (see MASKED for the report of a prior study using this measure). The interview takes approximately 1 h to complete and is videotaped and transcribed for coding purposes. Questions are coded using the manual, which provides examples of different types and levels of RF responses ranging from −1 (avoidance or active refusal to mentalize) to 9 (exceptionally rich, complete, and sophisticated understanding of the links between mental states and behaviors in interactions), where a 5 (clear and solid mental states understanding) is the mean observed in middle-class community samples. In this study, we explored maternal RF regarding own abuse experience among CSA-exposed mothers. All protocols were coded by two coders who were trained to code parental RF. Inter-rater reliability was calculated on 20% of protocols and was satisfactory (ICCs ranged from 0.67 to 0.98 and reached 0.93 for parental RF).

### Data Analytic Plan

Prior to testing study hypotheses, we first examined bivariate associations between demographic variables and key study variables with the intention of including as covariates any demographic variables significantly associated with dependent variables at the bivariate level. To examine the association between maternal and child CSA histories (both two level categorical variables), Pearson Chi-Square Analysis was used. To test the hypothesis involving moderation, we used PROCESS (Hayes, 2012) Model 1 (two-way interaction). PROCESS probes simple slopes of interaction effects using 1,000 bias-corrected, bootstrapped samples to estimate 95% confidence intervals, estimating associations between *x* and *y* at mean, one standard deviation below, and one standard deviation above mean levels of the moderator. To test Hypothesis 3, we used analyses of covariance (ANCOVAs) in which we entered the abuse group as the dependent variable and the clinical symptom category as the dependent variable. Due to the fact that we had so few children in one of our abuse groups (direct CSA exposure but no indirect CSA exposure, *n* = 6), we first conducted the ANCOVAs excluding children belonging to this group; then we conducted a second series of analyses including the full sample, results that we present for exploratory purposes only.

## Results

Descriptive statistics on the sample are provided in Table 1 and abuse sub-groupings are provided in Table 2. Zero-order correlations, presented in Table 3, revealed that both maternal and child CSA were significantly positively associated with children’s psychopathology; further, higher household income was significantly associated with lower likelihood of child and mother sexual abuse. Maternal and child abuse were positively intercorrelated. Since only household income was associated with dependent variables, we only controlled for this in analyses.

**TABLE 2:** Breakdown of sample into abuse subgroupings.

		Direct CSA Exposure		
		Child no CSA	Child CSA	Total
Vicarious CSA Exposure	Mother No CSA	42	6	68
	Mother CSA	26	37	43
	Total	63	48	111

*Direct CSA exposure refers to whether or not the child him/herself has been sexually abused, whereas vicarious CSA exposure refers to whether or not the child’s mother has been sexually abused as a child.*

**TABLE 3:** Zero-order correlations of key study variables.

	2	3	4	5	6
(1) Mother abuse	$0.47^{**}$	-0.03	$-0.34^{**}$	$0.45^{**}$	$0.38^{**}$
(2) Child abuse	—	0.003	$-0.23^{*}$	$0.40^{**}$	$0.40^{**}$
(3) Child age		—	0.11	0.05	-0.02
(4) Household income			—	-0.15	-0.16
(5) Internalizing				—	$0.69^{**}$
(6) Externalizing					—

*p < 0.05, ^∗∗^*p* < 0.01.

### Hypothesis Testing

**Hypothesis One. Are children whose mothers have experienced CSA more likely to themselves experience CSA?** A 2 x 2 Chi-square analysis revealed that maternal CSA exposure was significantly associated with likelihood of children’s CSA exposure, $\chi {}^{2}$(1) = 24.53, *p* < 0.001; children whose mothers had experienced CSA were more likely to also report CSA.

**FIGURE 1:**
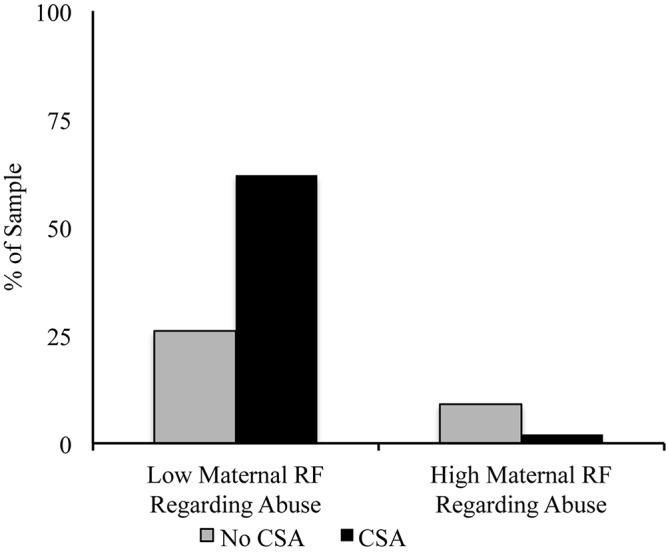
The percentage of children whose mothers experienced CSA who do and do not report CSA experiences as a function of maternal RF regarding own CSA experiences. For ease of interpretation, we depict values here as a function of whether or not maternal RF constituted mentalizing (scores of 5 higher), but the statistical analysis was conducted using continuous PDI RF scores.

**Hypothesis Two. Among CSA-exposed mothers, does maternal RF regarding own abuse predict lower likelihood of CSA in children?** A logistic regression analysis revealed that controlling for household income, maternal RF regarding own CSA significantly altered the odds ratio in the prediction of children’s CSA, $\chi {}^{2}$(1) = 5.11, *p* = 0.02. As maternal RF regarding own abuse increased, the odds of children’s CSA-exposure decreased, β = −0.30, *p* = 0.036 (see Figure 1). As a point of comparison, when we conducted the regression using parental RF instead of mothers’ RF regarding own abuse, RF did not alter the odds ratio, $\chi {}^{2}$(1) = 0.23, *p* = 0.63.

**FIGURE 2:**
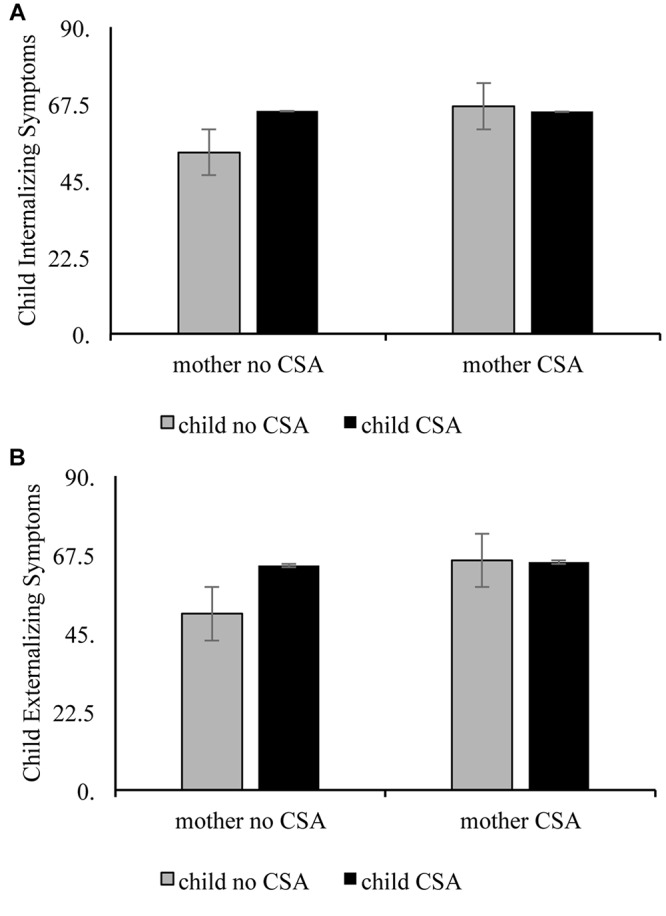
Child and maternal abuse history as interactive predictors of child internalizing**(A)** and externalizing**(B)** symptoms.

**Hypothesis Three. Are children’s psychological symptoms related to their own and their mothers’ history of CSA?** First, we explored abuse history as a predictor of children’s internalizing symptoms. As stated above, first we conducted the analysis including only the three groups of children with *n*’s over 25. The results of an ANCOVA controlling for household income revealed that abuse group was significantly associated with children’s internalizing symptoms, *F*(3,100) = 16.53, ´η^2^ = 0.26. The results of Least Significant Differences *post hoc* tests revealed that children with no direct or vicarious CSA had significantly lower internalizing symptoms than both other groups, *p*’s < 0.001. Children with no direct but with vicarious CSA exposure did not differ from children with direct and vicarious CSA exposure in internalizing symptoms. A follow-up analysis in which we included the six children with direct CSA exposure only revealed a similar pattern of effects to the first analysis; *F*(4,106) = 10.95, ´η^2^ = 0.25. In addition to the subgroup differences emerging in the first analysis, this ANCOVA also revealed that direct CSA only children had significantly higher internalizing than children with no CSA exposure, *p* = 0.006, but did not differ significantly from the two other abuse groups (see Figure 2A).

Next, we tested abuse history as a predictor of children’s externalizing symptoms. The results of an ANCOVA controlling for household income revealed that abuse group was significantly associated with children’s externalizing symptoms, *F*(3,100) = 11.14, ´η^2^ = 0.19. This revealed a similar pattern as above, with children with vicarious CSA exposure only not different significantly from children with both direct and vicarious, *p* = 0.11, and children with no CSA exposure having significantly lower externalizing than children with vicarious only, *p* = 0.009 or both direct and vicarious CSA-exposure, *p* < 0.001. A follow-up analysis in which we included the six children with direct CSA exposure only revealed a similar pattern of effects to the first analysis; *F*(4,106) = 8.28, ´η^2^ = 0.20. In addition to the subgroup differences emerging in the first analysis, this ANCOVA also revealed that direct CSA only children had significantly higher externalizing than children with no CSA exposure, *p* = 0.007, but did not differ significantly from the two other abuse groups (see Figure 2B).

## Discussion

Our central goal was to explore the interrelations between maternal CSA, children’s CSA, maternal T-RF, and children’s psychological adjustment in order to advance our understanding regarding intergenerational patterns of risk and the factors that may attenuate or heighten these cycles. Our findings provided mixed support of our hypotheses.

In line with previous findings (Zuravin et al., 1996; Avery et al., 2002; Lev-Wiesel, 2006; Testoni et al., 2018), we found that children of mothers who have experienced CSA have a higher likelihood of experiencing CSA themselves (Hypothesis 1). We believe this is the first study to document these effects among French Canadians, which we interpret to suggest that the pattern that has been observed in other cultural groups is also present among this population.

Although children of mothers with CSA were more likely to experience CSA themselves, rates of children’s CSA exposure were attenuated among dyads in which mothers demonstrated high levels of T-RF regarding their own abuse (Hypothesis 2). In other words, mothers who were able to speak in greater depth about their thoughts and feelings related to their own CSA exposure were less likely to have children who had been exposed to CSA. To our knowledge, no studies to date have examined T-RF as a protective factor in the intergenerational transmission of CSA; as the current study is cross-sectional, we cannot ascertain whether maternal T-RF has a protective influence, but documenting cross-sectional associations is an important first step in this direction. Theoretically, it makes sense to conceptualize maternal T-RF as having a protective role – perhaps thoroughly processing the emotions associated with one’s own CSA, as facilitated via mentalizing, can offset deleterious psychological, behavioral, or socioeconomic impacts associated with CSA that put the next generation at increased risk. These mothers may have fewer blind spots that can enhance risk for revictimization of oneself or one’s child (Testoni et al., 2018), which would underscore the public health significance of T-RF in the intergenerational transmission of abuse. Alternatively, mothers who are better able to mentalize about their experiences of abuse may have had other psychological resources that helped them to cope following CSA, such as more psychologically available caregivers, better pre-CSA mental health, or access to mental health services. While the cross-sectional nature of our data precludes our ability to identify whether T-RF plays a role in actively preventing children’s CSA exposure (i.e., it could be that CSA exposure in one’s child leads to decreases in mothers’ trauma-RF regarding their own CSA), these findings provide tantalizing support for the argument that mentalization-based treatments (e.g., Suchman et al., 2018) for CSA-exposed mothers may be able to protect their children against similar adversity.

Additionally, children with direct and vicarious (via their mothers) exposure to CSA were at greater risk for both internalizing and externalizing pathology (Hypothesis 3). In fact, our findings suggest that either direct or vicarious exposure to CSA is associated with more psychopathology symptoms, but that the addition of CSA from another source is not associated with worse symptoms. Specifically, in many of our analyses, CSA-exposed and CSA-non-exposed children whose mothers had been exposed to CSA did not differ in terms of symptoms. Similarly, mothers’ CSA history was associated with more symptoms in children only when children themselves had not been abused. We discuss the implications of each of these aspects of the findings in turn.

These findings and the design of the study do not enable us to identify mechanisms underlying these associations. However, we speculate that there are many possible mechanisms that could explain the risk associated with CSA to children or mothers. Maternal CSA may interfere with a variety of protective caregiving behaviors (Burkett, 1991; Cohen, 1995; Lyons-Ruth and Block, 1996; Zuravin et al., 1996), potentially due to the disruption of emotion regulation, a central psychological capacity in parenting (Rutherford et al., 2015); in turn, low levels of protective caregiving could be associated with psychopathology in children, thereby increasing children’s risk for psychopathology even in the absence of their own exposure to CSA. Mothers’ exposure to CSA could lead to “ghosts in the nursery,” or situations in which the child’s vulnerability evokes emotions related to mothers’ own abuse, resulting in fear, disorientation, or agitation in the children (Fraiberg et al., 1975; Lieberman and Van Horn, 2005; Karr-Morse and Wiley, 2007). Indeed, a body of research has examined parents’ unresolved trauma reactions as predictors of parenting behavior and children’s outcomes, with findings suggesting that trauma is associated with disruptions in caregiving behavior that could be frightening to children (Lyons-Ruth and Block, 1996; Schuengel et al., 1999).

That children exposed to CSA had higher psychopathology is consistent with the results of many prior studies finding that CSA-exposed youth are at risk for psychopathology (Kendall-Tackett et al., 1993; Maniglio, 2009; Mathews et al., 2013), even as compared to children who have experienced other forms of abuse (Fergusson et al., 2008; Lewis et al., 2016). Here, too, the mechanisms underlying these associations are not explicated through the current study, but prior work has suggested that exposure to CSA could adversely impact stress reactivity and generate feelings of self-blame or loneliness (Metzer et al., 1999; Filipas and Ullman, 2006; Murphy et al., 2013), which in turn may confer risk for psychopathology. Our theoretical model holds that CSA heightens risk for psychopathology; however, given that the current investigation was cross-sectional, we cannot ascertain whether CSA preceded or followed symptomatology. Socially isolated children are more likely to be victimized by sexual predators (Boney-McCoy and Finkelhor, 1996; Fleming et al., 1997), and given that socially isolated children have more symptoms of psychopathology (Rubin and Mills, 1988; Qualter and Munn, 2002; Hall-Lande et al., 2007), it is possible that psychopathology precedes CSA exposure, or that there are bidirectional pathways between the two.

It is also worth noting that among children whose mothers had experienced CSA, children’s exposure to CSA was not associated with greater symptoms. This is somewhat surprising in that we would have expected to find evidence for a model of cumulative risk (Appleyard et al., 2005; Kraemer et al., 2005; Chartier et al., 2010), wherein children’s CSA exposure would intensify the adverse outcomes of having a mother who has experienced CSA. Perhaps mothers who have experienced CSA are better equipped than mothers who have not experienced CSA to support their children following children’s CSA exposure – having been through CSA themselves, they may have guidance to offer their children in handling this experience. Or perhaps children’s CSA exposure enhances feelings of closeness or connection between mothers and children in a way that protects children. Another explanation is that the children of CSA-exposed mothers have developed in the context of intergenerational abuse, which leads them to have an internal working model (IWM) that is relatively adaptive in the face of abuse. In other words, children of CSA-exposed mothers may be more “prepared” for CSA than children whose mothers have not been abused. Though controversial, this perspective is in line with the core tenets of attachment theory (Bowlby, 1973), which states that children’s IWMs of attachment develop in adaptation to the context in which they are raised. In this sense, experiences that violate the expectations of the IWM (particularly those that deviate the IWM’s assumptions by being unexpectedly negative) would be more disruptive to the individual than experiences that confirm the IWM’s assumptions. This reasoning is consistent with recent work suggesting that adults with high attachment anxiety and high trauma exposure exhibit better psychological adjustment than those with low attachment anxiety and high trauma exposure following the conclusion of a stressful life event (end of a military deployment; Borelli et al., 2018b) – in this study, the authors argued their findings may provide evidence of the adaptive nature of attachment insecurity in the face of stress. In other words, although insecure or disorganized attachment in and of itself is associated with negative mental health outcomes in children (see Groh et al., 2012 for a review; Mikulincer and Shaver, 2012), perhaps insecure IWMs protect children from the maladjustment that could stem from their own abuse – if they are expecting others to harm or frighten them, then CSA exposure could be less “disruptive” in terms of shifting children’s world views. Indeed, the Just World Hypothesis (Lerner and Miller, 1978) holds that one of the most upsetting aspects of trauma exposure is the loss of control and of the conviction that the world is a safe place where good things happen to good people; children with insecure/disorganized attachment likely do not have that perspective to begin with, which could mean that in some sense they are more “prepared” for cataclysmic events such as CSA exposure.

### Implications

If replicated using longitudinal designs, the results of this study would underscore the importance of mothers’ mentalization regarding their own CSA (when relevant), and the potential utility of family-based approaches to the treatment of sexual trauma. After the initial shock of trauma, treatment all too often ignores the traumatic shrapnel – indirect psychological wounds that affect the entire family system as well as wounds that have impacts long after the immediate threat is gone (Testoni et al., 2018). The results of this paper suggest that children whose mothers have endured CSA are more likely to have psychopathology regardless of whether they have experienced CSA, a finding echoed in studies demonstrating similar connections between maternal CSA and child outcomes that have not accounted for children’s CSA-exposure (e.g., Burkett, 1991; Leifer et al., 2004). Our study also suggests that longitudinal studies should explore RF regarding one’s own CSA as a potential protective factor against the next generation’s CSA-exposure. Focusing on the recognition and understanding of one’s own and others’ emotions has been shown to help aid healthy reflection and enhance emotion regulation (Fonagy et al., 1991, 2002, 2003).

Importantly, we believe that our findings underscore a point that others have asserted (e.g., Testoni et al., 2018) – namely, the notion that people exposed to CSA may be more likely to be exposed to situations that can result in their own revictimization or in the victimization of their children. Further, our findings suggest that perhaps even when the children of CSA-exposed mothers are not exposed to CSA themselves, they nonetheless have higher psychopathology symptoms. It may be important to direct additional mental health services toward CSA-exposed adults and their children, both as a means to prevent an intergenerational cycle of CSA and to improve mental health.

### Limitations and Future Directions

The study has a number of strengths, including the use of an interview measure to evaluate RF and the focus on a difficult-to-recruit but important population. The children in this study experienced a range of types and levels of CSA seen in a community sample, but which may still be different from the experiences of children put into the care of child protective services, such as severe, chronic CSA together with neglect, physical abuse, and emotional abuse (Robbie Rossman et al., 1998; English et al., 2005; Lau et al., 2005). Research on the associations of maternal RF within different samples of children with CSA is thus warranted. Additionally, this is the first study to our knowledge to examine the role of RF in the interactive effect of mother and child CSA on child psychopathology.

Other limitations of this study warrant discussion and should be addressed in future investigations. The cross-sectional nature of the study limits the extent to which a temporal developmental sequence can be inferred. Furthermore, while children’s CSA was measured using an impartial, multi-reporter approach, mother’s CSA was measured retrospectively. This methodology introduces the possibility of recall bias that could confound our findings – specifically, adults who struggle with adjustment may report more instances of abuse (Fisher et al., 2009; Sheikh et al., 2016a, b). We also gathered little information regarding the nature of mothers’ experiences of CSA. Future studies should collect data on the details of both mothers’ and children’s abuse in order to better understand detailed intergenerational connections and to consider maternal T-RF in the context of the severity of mothers’ abuse experiences. Additionally, our hypotheses were examined using an ethnically homogeneous sample from North America. To increase the generalizability of our findings, studies exploring these same research questions should be conducted in more diverse samples.

Future studies may also wish to examine the impact that other trauma reactions may have on maternal T-RF and other aspects of trauma processing. Schechter et al. (2005) posit that for mothers with post-traumatic stress disorder (PTSD) symptoms, feelings of anger or shame surrounding their trauma experience may negatively color the way they view their child independently of RF abilities. In this sample, mothers with both high levels of PTSD symptoms and of RF still had distorted mental representations about their child, potentially resulting in hostile attributions toward their child, even in mothers with higher levels of RF. Additionally, mothers with trauma histories may benefit from psychologically distancing themselves from their children’s trauma experiences as a coping mechanism in order to prevent feelings of re-traumatization; several studies have shown that individuals tend to demonstrate greater psychological distance when discussing traumatic events (Koss and Kilpatrick, 2001; Pennebaker, 2003; Cohn et al., 2004). It may be useful to investigate links between T-RF and trauma-related psychopathology for potential impacts on mother and child trauma outcomes.

## Conclusion

Our findings uncover nuanced links between CSA, T-RF, and psychopathology, with implications for the potential importance of mentalization- and family-based approaches to the treatment and prevention of sexual trauma. Conceptualizing healing through a holistic lens means understanding family members’ roles in the process, whether it be supporting the victim, or understanding individual trauma reactions and histories. We find that the link between CSA exposure and children’s outcomes is more complex than it may seem, and that in particular understanding the roles of maternal CSA-exposure and T-RF may help enhance the development of models of risk and resilience.

## Ethics Statement

All subjects gave written informed consent (mothers) and informed assent (children) in accordance with the Declaration of Helsinki. The protocol was approved by the Institutional Review Board at Universite Laval.

## Author Contributions

JB conceived the study design, conducted all analyses, and was the author primarily responsible for the writing of the manuscript. CC assisted with the study design and analyses and contributed to the writing of the manuscript. CP assisted in the interpretation of the findings and contributed to the writing and editing of the manuscript. LN, MT, and PF provided feedback on the study design and conceptualization, as well as read and commented on the manuscript draft. KE contributed to the development of the research questions and the manuscript writing.

## Conflict of Interest Statement

The authors declare that the research was conducted in the absence of any commercial or financial relationships that could be construed as a potential conflict of interest.
